# Quality of life in head and neck cancer patients at 5 years after free flap reconstruction: a significant decline during the follow-up

**DOI:** 10.1007/s00405-021-07242-1

**Published:** 2022-01-05

**Authors:** Sanna Lahtinen, Krisztina Molnár, Siiri Hietanen, Petri Koivunen, Pasi Ohtonen, Antti Alakärppä, Janne Liisanantti

**Affiliations:** 1 Department of Anesthesiology, Oulu University Hospital (OYS), P.O. BOX 21, 90029 Oulu, Finland; 2 Research Group of Surgery, Anesthesiology and Intensive Care, MRC Oulu, Oulu, Finland; 3 Department of Otorhinolaryngology and Head and Neck Surgery, Oulu University Hospital, Oulu, Finland; 4 Department of Operative Care, Oulu University Hospital, Oulu, Finland; 5 PEDEGO Research Unit, MRC Oulu, Oulu, Finland

**Keywords:** Cancer of the head and neck, Free flap surgery, Quality of life, Long-term follow-up

## Abstract

**Purpose:**

Free flap reconstructions following head and neck tumor resection are known to involve more than 50% rate of complications and other adverse events and up to 50% mortality during a 5-year follow-up.

We aimed to examine the difference in the long-term quality of life (QoL) between the 2-year and 5-year assessments after free flap surgery for cancer of the head and neck.

**Methods:**

A total of 28 of the 39 eligible patients responded to the survey. QoL was assessed at 5 years after operation and compared with the assessment performed at 2 years after the operation using RAND-36, EORTC-C30 and H&N-35, and SWAL-QOL tools.

**Results:**

The criteria for poor QoL using RAND-36 tool was met in 11 (39.3%) patients in contrast to 4 (14.3%, *P* = 0.003) patients in the 2-year assessment. EORTC-C30 global score was decreased from 83.9 (SD16.4) to 64.6 (SD 24.0, *P* < 0.001) during the follow-up. In both RAND-36 and EORTC-C30 surveys, decline was found in physical and role functioning together with energy and emotional well-being domains. SWAL-QOL showed poor swallowing-related QoL in both assessments.

**Conclusion:**

We found a significant decline in QoL during a 5-year follow-up after free flap surgery for cancer of the head and neck.

## Introduction

Free-flap reconstructions following head and neck tumor resection are known to involve more than 50% of complication and other adverse events rate, and up to 50% mortality during a 5-year follow-up [1, 2]. The most dominant factors affecting the long-term outcome are patient related, such as postoperative medical complications and comorbidities [1, 2]. Especially, postoperative medical complications, including pneumonia, myocardial infarction or stroke have negative impact on both long-term outcome and quality of life (QoL) [2, 3].

It is previously demonstrated that QoL assessed with RAND-36 tool 2 years after the free flap operation is comparable with QoL of general population in patients without postoperative complications [3]. Other studies using different tools, such as EORTC QLQ-C30 have shown similar results [4–6]. Both patient- and treatment-related factors have shown to be linked to the assessed QoL [5, 6]. According to our knowledge, most of the previous studies have reported the evaluations only up to 2 years after the operation and the number of studies focusing on the QoL in a longer follow-up is limited. In one Finnish survey, the QoL was assessed with 15D-tool and the study showed a reduction in the QoL during a 5-year follow-up [7].

We determined to examine the QoL at 5 years after the primary operation and to compare the measured QoL to the assessment performed at 2 years after operation using RAND-36-, EORTC-C30-, EORTC-HNC35-, and SWALQ-tools. In addition, we wanted to examine the impact of the recorded postoperative complications on the long-term QoL.

## Materials and methods

This prospective cross-sectional cohort study was conducted one of the five University Hospitals in Finland, providing tertiary care for patients undergoing free flap surgery due to cancer of the head and neck. The study protocol was accepted by the hospital administration (239/2016) and local ethics committee (The Regional Ethics Committee 95/2016).

### Patients

The original cohort consisted of all the 54 2-year survivors operated with free flap reconstruction during the period 2013–2016. One patient was excluded for not being eligible (3). The patients’ QoL was assessed at 2 years after the operation during the period 2017–2018 (baseline assessment). Of the original cohort, 39 patients were alive, and 28 patients responded to the long-term QoL assessment at 5 years after the operation. Two patients were in palliative care, and therefore, not contacted, and nine patients did not respond to the survey.

### Assessments

Baseline assessment was performed at 2 years after the operation either by interview during normal control visit or by letter. For the 5-year assessment, the patients alive were contacted by telephone or SMS, after which the questionnaires were sent to them. RAND-36, EORTC-QLQ-C30, EORTC-QLQ-H&N-C35 and SWAL-QOL- questionnaires were used to assess the QoL. Beck depression inventory (BDI) was used to assess depression [8]. In the BDI score of 7 or more was considered clinically significant.

#### RAND-36

RAND-36 is a general measure of health and well-being consisting of eight domains (general health, physical functioning, physical and emotional role functioning, emotional well-being, social functioning, pain, energy/fatigue) scored in a scale from 0 (the worst) to 100 (the best) [9]. There are Finnish age-adjusted RAND-36 reference values available and these were used for comparison [10]. Poor QOL in each domain was determined as a difference of more than − 2 SD compared with the age-adjusted reference values. The overall QoL was considered poor if one or more of the domains met the criteria.

### EORTC-QLQ-C30 & EORTC QLQ-H&N35

EORTC QLQ-C30 is a questionnaire used for evaluating the general QOL among cancer patients. The questionnaire consists of five functioning scales, three symptom scales, a global QOL scale, and six single items assessing other symptoms and problems often reported by cancer patients. EORTC QLQ-H&N35 is a tumor-specific QOL questionnaire specially designed for head and neck cancer patients. This questionnaire includes seven symptom scales, six single items, and five optional items that evaluate the impact of tumor location and treatment on QOL. The scales and single-item scores of both EORTC instruments are linearly transformed into a score of 0–100. A high scores from the functional scale and the global QOL scale represents a high level of functioning, whereas a high score from the symptom scale or a single item represents a high level of symptoms [11, 12].

#### SWAL-QOL

SWAL-QOL consists of 44 questions assessing ten QOL domains: food selection, burden, mental health, social functioning, fear, eating duration, eating desire, communication, sleep, and fatigue. Sleep and fatigue contribute to general QOL, whereas the other domains are contributors to dysphagia-specific QOL. For analyzing the results, each scale is constructed using Likert`s method, which equally weighs each item and sums them into an overall scale score. All scales are transformed to a 0–100 metric, 100 indicating the most favorable state and 0 the least favorable, and scores in between representing the percentage of the total possible score achieved. SWAL-QOL is developed to assess the impact of oropharyngeal dysphagia in neurologic diseases but has also been used in other conditions causing swallowing problems, i.e., head and neck cancer. The total SWAL-QOL score includes 23 items from 7 domains (communication, sleep and fatigue excluded). A cut-off value of 86 is used to determine significant impairment of the swallowing-related QOL [13].

### Statistical analysis

Statistical analysis was performed using SPSS for Windows (IBM Corp., IBM SPSS Statistics for Windows, version 25, Armonk, NY, USA). Categorical variables are presented as number (*n*) and percentage (%) and continuous variables in mean and standard deviations (SD). Differences between the QoL assessments were tested using paired samples *t* test. Continuous variables were tested using Mann–Whitney test and categorical values using Fisher’s exact test. *P* value less than 0.05 was considered statistically significant.

## Results

The QoL assessment was performed at 5.3 (SD 0.9) years from the operation and mean time between the assessments was 2.9 (SD 0.5) years. The criteria for poor QoL was met in 11 (39.3%) patients. In the baseline assessment the criteria for poor QoL was met in 4 (14.3%, *P* = 0.003) patients. Patients with poor QOL were older compared with those with good QoL. There were no differences between groups in other demographic factors (Table 1).

**Table 1:** Patient characteristics and quality of life assessments of the 28 patients operated with free flap reconstruction for cancer of the head and neck

	Interviewed *n* = 28	Good QoL	Poor QoL	*P* value
		*N* = 17	*N* = 11	
Age at assessment (y)	68.3 (9.7)	66.4 (9.0)	71.2 (10.5)	0.047
Perioperative data				
Gender f/m	19/9	12/5	7/4	0.507
ASA				
1–2	17 (60.7)	12 (70.6)	5 (45.5)	0.175
3–4	11 (39.3)	5 (29.4)	6 (54.5)	
CCI > 1	11 (39.3)	5 (29.4)	6 (54.5)	0.175
Smoking	11 (39.3)	8 (47.1)	3 (27.3)	0.260
Alcohol abuse	3 (10.7)	1 (5.9)	2 (18.2)	0.336
BMI	25.1 (5.5)	24.6 (6.3)	25.9 (4.4)	0.352
Tumor				
Oral cavity/tongue	12 (42.9)	8 (47.2)	4 (36.4)	
Maxilla	4 (14.3)	1 (5.9)	3 (27.3)	
Mandibula	3 (10.7)	1 (5.9)	2 (18.2)	
Larynx/pharynx	1 (3.6)	1 (5.9)	0	
Palatinal	4 (14.3)	2 (11.8)	2 (18.2)	
Buccal mucosa	3 (10.7)	3 17.6)	0	
Others	1 (3.6)	1 (5.9)	0	
Free flap				
RFA	14 (50.0)	7 (41.2)	7 (63.6)	
ALT	2 (7.1)	2 (11.8)	0	
Scapula	1 (3.6)	0	1 (9.1)	
Fibula	4 (14.3)	4 (23.5)	4 (14.3)	
Lateral arm	6 (21.4)	4 (23.5)	2 (18.2)	
Crista	1 (3.6)	0	1 (9.1)	
Tracheostomy	23 (82.1)	16 (94.1)	8 (72.7)	0.157
Neck dissection	22 (78.5)	14 (82.4)	8 (72.7)	0.439
Complication	12 (42.9)	9 (52.9)	3 (27.3)	0.172
Surgical complication	11 (39.3)	8 (47.1)	3 (27.3)	0.260
Medical complication	6 (21.4)	4 (23.5)	2 (18.2)	0.561
Baseline assessment				
Poor baseline QoL	4 (14.3)	1 (6.7)	3 (23.1)	0.09
EORTC global score	83.9 (16.3)	84.3 (17.6)	83.3 (14.9)	0.209
6 min walking test*	101.2 (16.0)	105.0 (17.6)	95.9 (12.6)	> 0.9
Hand grip test	29.2 (12.3)	28.9 (10.1)	29.6 (29.2)	0.561
5-year assessment				
Time from the operation, years	5.3 (0.9)	5.3 (0.9)	5.0 (0.9)	0.429
Time between the assessments	2.9 (0.5)	3.0 (0.5)	2.7 (0.4)	0.331
EORTC global score	64.6 (24.0)	71.2 (25.5)	54.5 (18.0)	0.07
SWQL < 86, data missing 5	18 (78.3)	10 (76.9)	8 (80.0)	0.633
BDI score > 7	14 (53.8)	8 (50.0)	6 (60.0)	0.464
BDI	10.4 (8.2)	8.3 (6.8)	13.8 (9.7)	0.317

*Percentages of age-adjusted normal score

### RAND-36

The comparison between the 2-year and 5-year evaluations showed significant reduction in all the RAND-36 domains except role functioning. The measured RAND-36 values were lower than the general population values in all the domains except energy/fatigue (Table 2). Postoperative complications did not have an impact on the RAND-36 assessments at 5 years after the operation (Table 3).

**Table 2:** Differences in RAND-36 dimensions between assessments at 2 years and 5 years after free flap surgery for cancer of the head and neck

Dimension	5-year value	Baseline	Difference	*P* value	General value	Number of responders answering below age-adjusted reference
Physical functioning	68.0 (27.0)	86.1 (21.8)	− 18.0 (− 25.5 to − 10.5)	< 0.001	84.9 (22.1)	12 (35.7)
Role functioning physical	58.3 (41.6)	76.4 (27.8)	− 18.1 (34.3 to − 1.8)	0.031	74.8 (35.5)	10 (35.7)
Role functioning emotional	65.4 (42.8)	79.0 (30.9)	− 13.6 (31.6 to − 4.4)	0.133	75.0 (36.4)	13 (16.4)
Energy/fatigue	60.4 (23.9)	78.9 (14.9)	− 18.5 (25.7 to − 11.3)	< 0.001	64.0 (22.4)	10 (35.7)
Emotional well-being	75.1 (17.5)	85.8 (11.4)	− 10.7 (17.3 to − 4.0)	0.003	73.7 (19.7)	11 (39.3)
Social functioning	70.1 (26.9)	85.3 (18.3)	− 15.2 (27.2 to − 3.1)	0.015	82.1 (23.2)	16 (57.1)
Pain	66.6 (25.5)	81.1 (19.3)	− 14.5 (− 23.9 to − 5.0)	0.004	76.2 (24.0)	13 (46.4)
General health	52.2 (21.8)	63.1 (19.9)	− 10.9 (− 17.9 to 3.9)	0.003	65.0 (19.8)	10 (35.7)

**Table 3:** RAND-36 values of 28 patients with and without complications after free flap surgery for cancer of the head and neck in comparison with RAND-36 values of general Finnish population

	Complications	No complications	*P* value	General population
Physical functioning	63 3 (27.0)	71.6 (27.4)	0.397	84.9 (22.1)
Role functioning physical	72.7 (30.5)	48.4 (46.1)	0.195	74.8 (35.5)
Role functioning emotional	84.8 (27.3)	52.1 (47.1)	0.089	75.0 (36.4)
Energy/fatigue	64.1 (17.8)	57.8 (27.1)	0.610	64.0 (22.4)
Emotional well-being	76.4 (13.7)	74.3 (20.1)	0.981	73.7 (19.7)
Social functioning	67.7 (25.3)	71.9 (28.7)	0.599	82.1 (23.2)
Pain	63.1 (18.2)	69.2 (30.2)	0.371	76.2 (24.0)
General health	51.8 (25.8)	52.5 (19.5)	0.790	65.0 (19.8)

### EORTC-C30 & EORTC- H&N35

The global health status and all the functional scales declined significantly between the 2-year and 5-year assessments. There was increase in symptom scales of fatigue, pain, and diarrhea. Of the EORTC-H&N35 dimensions, there was increase in symptom scales of social eating, social contact, less sexuality, and felt ill. There was decrease in scales of pain killers and sensing problems (Table 4).

**Table 4:** Differences in EOTC-C30 and EORTC H&N35 dimensions between assessments at baseline and at 5 years after free flap surgery for cancer of the head and neck

Dimension	5-year value	Baseline	Difference	*P* value
EORTC-C30				
Global health status	64.6 (24.0)	83.9 (16.4)	− 19.3 (− 27.6 to 11.07)	< 0.001
Functional scales				
Physical functioning	71.4 (22.6)	86.0 (18.2)	− 14.6 (− 11.0 to − 8.6)	< 0.001
Role functioning	82.1 (22.2)	91.1 (18.4)	− 8.9 (17.8 to − 0.05)	0.049
Emotional functioning	84.2 (16.3)	90.5 (11.5)	− 6.3 (-12.0 to − 0.5)	0.034
Cognitive functioning	81.5 (21.9)	90.5 (11.5)	− 8.9 (− 15.1 to − 2.7)	0.007
Social functioning	81.0 (18.6)	89.9 (12.3)	− 8.9 (19.3 to − 1.4)	0.087
Symptom scales				
Fatigue	30.2 (20.5)	13.1 (15.7)	17.1 (11.0 to 23.1)	< 0.001
Nausea and vomiting	4.5 (11.9)	1.8 (5.2)	2.7 (− 1.6 to 7.0)	0.213
Pain	29.8 (23.3)	14.9 (16.6)	14.9 (7.3 to 22.4)	< 0.001
Dyspnea	15.5 (24.8)	10.7 (24.1)	4.8 (− 2.0 to 14.6)	0.161
Insomnia	28.6 (25.2)	19.0 (24.7)	9.5 (1.0 to 18.0)	0.030
Appetite loss	9.5 (20.0)	3.6 (10.5)	6.0 (− 2.7 to 14.6)	0.170
Constipation	13.1 (22.8)	8.3 (14.7)	4.8 (− 5.6 to 15.1)	0.355
Diarrhea	9.5 (17.8)	2.4 (8.7)	7.1 (0.7 to 13.6)	0.031
Financial difficulties	15.5 (24.8)	13.1 (22.8)	2.4 (− 6.9 to 11.6)	0.602
EORTC H&N35 symptom scales				
Pain	22.0 (20.2)	17.6 (17.9)	4.5 (1.0 to 9.9)	0.105
Swallowing	23.1 (23.4)	16.7 (21.8)	3.5 (− 4.1 to 11.2)	0.354
Senses problems	11.3 (20.8)	16.7 (21.8)	− 5.4 (− 10.0 to − 0.7)	0.026
Speech problems	27.2 (20.3)	20.2 (21.8)	7.0 (− 0.5 to 14.5)	0.067
Trouble with social eating	11.1 (21.2)	27.5 (27.5)	− 16.4 (–29.4 to -3 3)	0.016
Trouble with social contact	18.8 (16.9)	11.0 (13.2)	7.8 (3.3 to 12.4)	0.001
Less sexuality	51.3 (41.1)	26.7 (32.6)	24.7 (7.7 to 41.6)	0.006
Teeth	29.8 (35.5)	29.8 (37.7)	0 (− 18.6 to 18.6)	> 0.9
Opening mouth	39.3 (37.7)	32.1 (34.5)	7.1 (− 5.7 to 20.0)	0.264
Dry mouth	39.3 (32.8)	44.0 (34.0)	− 4.7 (− 18.3 to 8.7)	0.475
Sticky saliva	32.1 (34.5)	23.8 (31.2)	8.3 (− 4.2 to 20.8)	0.183
Coughing	21.4 (25.4)	9.5 (17.8)	11.9 (2.5 to 21.4)	0.015
Felt ill	23.8 (25.4)	6.0 (17.8)	17.9 (8.9 to 26.8)	< 0.001
Pain killers	16.7 (17.0)	35.7 (48.9)	− 19.0 (− 36.1 to − 2.0)	0.030
Nutritional supplements	13.1 (16.6)	25.0 (21.0)	− 11.9 (− 28.9 to 5.0)	0.161
Feeding tube	10.7 (31.5)	3.6 (10.5)	7.1 (1.0 to 15.3)	0.083
Weight loss	3.6 (10.5)	3.6 (18.9)	0.0 (− 8.6 to 8.6)	1.0
Weight gain	4.8 (11.9)	17.9 (39.0)	− 13.1 (− 28.6 to 2.4)	0.094

### SWAL-QOL

There were no differences between the 2-year and 5-year SWAL-QOL- evaluations. The SWAL-QOL total score was 66.3 (Table 5).

**Table 5:** Differences in SWAL-QOL dimensions between assessments at baseline and at 5 years after free flap surgery for cancer of the head and neck

	5-year value	Baseline	Difference	*P* value
Burden	66.6 (31.5)	68.0 (34.2)	− 1.4 (− 19.5 to 16.6)	0.870
Eating duration	41.3 (23.6)	36.5 (31.2)	4.8 (− 11.5 to 21.1)	0.550
Eating desire	76.1 (24.5)	81.7 (23.9)	− 5.6 (− 19.5 to 8.3)	0.412
Symptom frequency	69.2 (21.0)	75.7 (15.1)	− 6.6 (− 17.2 to 4.1)	0.215
Food selection	69.3 (31.0)	75.7 (15.1)	− 10.2 (− 25.9 to 5.5)	0.191
Communication	94.6 (21.0)	74.0 (23.1)	− 12.4 (− 26.2 to 1.3)	0.075
Fear of eating	87.5 (18.3)	91.3 (13.2)	− 6.7 (− 16.6 to 3.0)	0.169
Mental health	74.5 (30.3)	80.6 (24.8)	− 6.1 (− 22.7 to 10.4)	0.451
Social eating	70.0 (39.3)	77.6 (26.8)	− 7.6 (− 26.8 to 11.6)	0.422
Fatigue	69.0 (24.9)	80.7 (17.1)	− 11.7 (− 19.3 to − 4.0)	0.005
Sleep	64.5 (26.1)	72.5 (29.1)	− 8.0 (− 26.5 to 10.6)	0.385
SWAL-QOL total	66.3 (22.0)	76.8 (17.2)	− 10.57 (− 22.9 to 1.7)	0.088

## Discussion

The main finding of the present study is a significant reduction in the quality of life during the 3-year follow-up discovered using the RAND-36 tool. The rate of respondents reporting poor QoL increased more than two-fold during the 3-year follow-up. In addition, a reduction was seen in functional scales assessed with EORTC QLQ-30 tool. The swallowing-related QoL did not change over the time, however, most of the dimensions were scored low at the baseline assessment. Finally, according to the BDI score more than half of the respondents had signs of depression, however, this was not associated with poor QoL.

There is a limited number of previous studies focusing on long-term QoL in head and neck cancer survivors operated with free flap reconstruction. The present results are in line with the results reported by relatively recent Finnish study with a narrower range of assessments [7]. The probable reason for limited number of studies is the high mortality in this patient group but also the small cohorts limiting the possibilities for long-term follow-up.

The significant reduction in QoL can be explained by several factors. First, the patients were approximately 3 years older than during the baseline assessment and thus it is likely that other comorbidities as well as aging may have had an impact on the measured QoL. It has been shown previously that high utilization of health care resources is related to poor QoL measured using RAND-36 tool [10] Second, the decline in the QoL could be explained by the disease progression; the 5-year mortality has been reported to be more than 50% in this patient group and majority of these patients die due to cancer of the head and neck [1, 2, 14]. However, the present study setting does not allow us to confirm this hypothesis. Third, in our previous assessment, the measured QoL was comparable with the general population values [3] and one could hypothesize that at the 2-year assessment the respondents reported good QoL simply due to satisfaction for surviving. At the 5-year assessment they have achieved a steady state in the recovery and factors having a negative impact on the QoL become more prominent. For instance, the swallowing-related QoL was comparable between the assessments but at 5 years from the operation, it might be more important. However, this study setting does not allow us to confirm this hypothesis.

The measured BDI scores in the current study population indicated high rate of depression. We also showed a significant decline in all used QoL tools in mental health dimensions. This supports our hypothesis that at the 5-year assessment the patients’ life has changed from the winning the disease to living with the disease with its limitations to the daily life. These include decline in dimensions focusing on the social life and physical functioning as well as experienced general health. In the 2-year assessment the recorded medical complications had a significant negative impact on QoL, while surgical complications did not have an impact on the QoL [3]. As the medical complications have been associated with the long-term mortality, it is obvious that these patients were not included in the present cohort [2]. Surgical complications are more often operation related and these are prone to recover. This explains the absence of impact of the postoperative complications on the long-term QoL.

### Clinical impact

The current study showed the significant reduction in QoL reported at 5 years after free flap surgery due to cancer of the head and neck. We have previously reported that chronic comorbidities as well as the medical complications were related to poor outcome. The medical complications were more common among those patients who had poor QoL at 2 years after the operation [2, 3]. The respondents of this study did not face the medical complications postoperatively and they reported good QoL at 2 years after the operation. Disease progression and ageing as factors having negative impact on the QoL are not controllable. Problems in social functions, such as eating and communication, as well as in mental well-being are important factors causing deterioration of the QoL. These patients could benefit from support in daily-life activities, for instance psycho-social support and dietician consultations during the follow-up. Moreover, peer support might be beneficial for the patients suffering from several symptoms.

### Limitations

This study was limited by the low number of patients. In the previous assessment, we were able to recruit nearly 100% of the operated patients, but in this survey, the participants represented 72.0% of the survivors, which could be considered as a limitation. Furthermore, we did not take into account other factors that may have had an impact on the QoL, such as comorbidities or other diseases requiring operative care. Also, the baseline screening of depression would have been interesting in terms of follow-up data. We did not include pre- and postoperative radiation into the analysis, which may have had an impact on the results, especially in the swallowing-related QoL. The number of patients receiving radiation therapy would have been modest since in our previous study the rate of radiation therapy was approximately 30% resulting in ten possible patients in this cohort [2]. Finally, the perioperative data were collected retrospectively, which can be considered as a limitation even though the operative care was provided at 5 years before the assessment and thus probably played a minor role in the current situation of the respondents.

## Conclusion

We found a significant reduction in QoL between the 2-year and 5-year assessments in patients who had been operated with free flap reconstruction due to cancer of the head and neck. The patients and treating physicians should be aware of possible treatment- and disease-related adverse effects in long-term follow-up. Decreases in mental health and social as well as physical functioning explained most of the decline in the quality of life. Intraoperative factors and postoperative complications did not have an impact on the measured QoL.
